# Assessing the Genotypic Differences between Strains of *Corynebacterium pseudotuberculosis* biovar *equi* through Comparative Genomics

**DOI:** 10.1371/journal.pone.0170676

**Published:** 2017-01-26

**Authors:** Rafael A. Baraúna, Rommel T. J. Ramos, Adonney A. O. Veras, Kenny C. Pinheiro, Leandro J. Benevides, Marcus V. C. Viana, Luís C. Guimarães, Judy M. Edman, Sharon J. Spier, Vasco Azevedo, Artur Silva

**Affiliations:** 1 Laboratory of Genomics and Bioinformatics, Center of Genomics and Systems Biology, Institute of Biological Sciences, Federal University of Pará, Belém, Pará, Brazil; 2 Laboratory of Cellular and Molecular Genetics, Institute of Biological Sciences, Federal University of Minas Gerais, Belo Horizonte, Minas Gerais, Brazil; 3 School of Veterinary Medicine, Department of Medicine and Epidemiology, University of California Davis, Davis, California, United States of America; University of Minnesota, UNITED STATES

## Abstract

Seven genomes of *Corynebacterium pseudotuberculosis* biovar *equi* were sequenced on the Ion Torrent PGM platform, generating high-quality scaffolds over 2.35 Mbp. This bacterium is the causative agent of disease known as “pigeon fever” which commonly affects horses worldwide. The pangenome of biovar equi was calculated and two phylogenomic approaches were used to identify clustering patterns within *Corynebacterium* genus. Furthermore, other comparative analyses were performed including the prediction of genomic islands and prophages, and SNP-based phylogeny. In the phylogenomic tree, *C*. *pseudotuberculosis* was divided into two distinct clades, one formed by nitrate non-reducing species (biovar *ovis*) and another formed by nitrate-reducing species (biovar *equi*). In the latter group, the strains isolated from California were more related to each other, while the strains CIP 52.97 and 1/06-A formed the outermost clade of the biovar *equi*. A total of 1,355 core genes were identified, corresponding to 42.5% of the pangenome. This pangenome has one of the smallest core genomes described in the literature, suggesting a high genetic variability of biovar *equi* of *C*. *pseudotuberculosis*. The analysis of the similarity between the resistance islands identified a higher proximity between the strains that caused more severe infectious conditions (infection in the internal organs). Pathogenicity islands were largely conserved between strains. Several genes that modulate the pathogenicity of *C*. *pseudotuberculosis* were described including peptidases, recombination enzymes, micoside synthesis enzymes, bacteriocins with antimicrobial activity and several others. Finally, no genotypic differences were observed between the strains that caused the three different types of infection (external abscess formation, infection with abscess formation in the internal organs, and ulcerative lymphangitis). Instead, it was noted that there is a higher phenetic correlation between strains isolated at California compared to the other strains. Additionally, high variability of resistance islands suggests gene acquisition through several events of horizontal gene transfer.

## Introduction

One of the main diseases that affects horses worldwide is popularly known as “pigeon fever”, and it is characterized by the formation of painful abscesses in the pectoral region resembling a pigeon’s breast and sometimes is accompanied by fever. The disease may occasionally be developed in two other forms, infection with purulent abscess formation in the internal organs, which is the most severe form of the disease, or ulcerative lymphangitis, which is the least frequent clinical form and is characterized by the infection of peripheral lymph vessels, severe swelling of the limbs and lameness [1].

The causative agent of the disease is *Corynebacterium pseudotuberculosis* biovar *equi*, a pleomorphic Gram-positive bacteria that, is facultative intracellular, and nitrate-reducing. The reservoir for infection is soil [2]. The transmission of disease occurs through contact with contaminated soil or pus from an infected horse via fly vectors, breaks in the skin or by inhalation. [3, 4]. *C*. *pseudotuberculosis* is also the causative agent of caseous lymphadenitis in small ruminants. However, the strains that cause this disease are grouped into another biovar called *ovis* [5]. At first, the distinction between the two biovars, *ovis* and *equi*, was based mainly on the nitrate reduction capability and results of restriction fragment length polymorphisms (RFLP) [6, 7].

Although the incidence of disease in horses suffers strong temporal variations, recent data showed an increase in the number of infections in the USA characterizing this species as a re-emergent animal pathogen [8, 9]. Geoclimatic conditions strongly influence the appearance of disease outbreaks, e.g., high surface land temperature (>35°C), dry soil, among other factors [10]. These geoclimatic characteristics indicated that outbreaks are favored in hot climate areas with low rainfall. In the USA, high incidence rates of the disease have been described in Texas [9], Colorado [11] California [12] and several other states [8]. This problem is not restricted to North America, however, and several other strains have been isolated from animals around the world, such as in Chile [13] and Egypt [14].

Until now, no phenotypic or genotypic characteristic of biovar *equi* could be related to the different forms of infection that can be caused by the pathogen. Britz and colleagues [15] evaluated phenotypic characteristics of equine isolates, such as reducing arabinose, sucrose, dextrin or nitrate, but the results showed no statistical correlation between these phenotypes and the location and extent of lesions caused by the bacteria. On the other hand, the genotypic characteristics can best be accessed and analyzed by sequencing the genome of the *equi* strains. Recently, several complete genomes of this biovar were published [16, 17] providing a large amount of biological data that can be used for comparative genomics studies.

These analyses have been widely employed in bacterial taxa with a large number of genomes sequenced to determine molecular features that contribute to the virulence and pathogenicity of these species [18, 19]. Comparative genomics enables the differentiation of genotypes based on the genome identity level, allows the characterization of pangenome and their subsets (shared and singletons genes), permits the identification of the evolutionary process (loss and gain of genes), and several other applications. Through comparative genomics, Soares and colleagues [19] identified significant differences in the composition of pathogenicity islands among biovars *ovis* and *equi* of *C*. *pseudotuberculosis* and demonstrated the high clonal behavior of strains that infect small ruminants and a greater plasticity in strains belonging to the biovar *equi*.

In this context, this study aimed to sequence and characterize the genomes of seven new strains of the biovar *equi*, isolated from horses diagnosed with *C*. *pseudotuberculosis* infection in the state of California, USA. A comparative genomic analysis was carried out with these seven genomes, together with twelve genomes of the biovar *equi* available in the National Center for Biotechnology Information (NCBI) database, totaling 19 genomes analyzed. Comparative analyses included: pangenome calculation of biovar *equi*; phylogenomic clustering of *Corynebacterium* genus; prediction of genomic islands (GEIs) and prophages; and SNP-based phylogeny.

## Results and Discussion

### Major genomic features

The main genomic features of the seven strains sequenced in this study are shown in Table 1. These characteristics are very similar to other strains of the same species previously sequenced that were isolated from different hosts such as horses [16] cows [20] and even humans [21]. All these strains showed a high GC content (~ 52%) and carried important virulence factors such as phospholipase D and pili-formation proteins, among other factors. After assembly, the contigs of the seven strains were ordered in a high-quality scaffold with the MAUVE program [22] using the MB11 strain as a reference because its genome order was validated with an optical map.

**Table 1:** Genomic features of the twelve strains isolated in California, including the seven strains described in this work.

Organism names	Date of isolation	Source of isolation	Genome size (bp)	Number of tRNAs	Number of CDSs	Number of rRNA clusters	Number of pseudogenes	Putative virulence factors	Putative resistance genes	Number of contigs	Reference
*C*. *pseudotuberculosis* MB44	Jun-01	Pectoral abscess	2,362,580	51	2,179	4	24	607	155	5	This article
*C*. *pseudotuberculosis* MB122	Mar-03	Liver, lung, kidney abscesses	2,374,496	51	2,152	4	79	592	154	3	This article
*C*. *pseudotuberculosis* MB154	Sep-03	Petoral, liver, lung and kidney abscesses	2,364,722	51	2,154	4	74	538	141	4	This article
*C*. *pseudotuberculosis* MB278	May-09	Renal abscess	2,369,798	51	2,204	4	27	610	161	3	This article
*C*. *pseudotuberculosis* MB295	Oct-09	Septic arthritis in hock	2,355,404	51	2,202	4	16	609	156	4	This article
*C*. *pseudotuberculosis* MB302	Nov-09	Liver abscess and abscess in tendon	2,370,427	51	2,164	4	64	595	149	4	This article
*C*. *pseudotuberculosis* MB336	Aug-12	Pectoral abscess	2,363,713	51	2,131	4	44	561	140	8	This article
*C*. *pseudotuberculosis* MB11	Oct-96	Ulcerative lymphangitis	2,363,423	51	2,169	4	37	598	152	1	CP013260
*C*. *pseudotuberculosis* MB14	Dec-96	Liver and kidney abscesses	2,370,761	51	2,235	4	20	609	153	1	CP013261
*C*. *pseudotuberculosis* MB30	Nov-00	Pectoral abscess	2,364,377	51	2,225	4	6	612	158	1	CP013262
*C*. *pseudotuberculosis* MB66	Jun-02	Kidney, liver and lung abscesses	2,372,202	51	2,201	4	54	597	151	1	CP013263
*C*. *pseudotuberculosis* MB20	Nov-97	Pectoral abscess	2,363,089	51	2,365	4	35	605	154	1	[16]

The number of tRNAs was determined with the tool tRNAscan-SE [23]. The numbers of CDSs, rRNA clusters, pseudogenes, and contigs were manually specified using the software Artemis v.14.0.0 [24]. The access numbers for each strain are: *C*. *pseudotuberculosis* MB44 (MCOC01000000), *C*. *pseudotuberculosis* MB122 (MCOB01000000), *C*. *pseudotuberculosis* MB154 (MCOA01000000), *C*. *pseudotuberculosis* MB278 (MCNZ01000000), *C*. *pseudotuberculosis* MB295 (MCNY01000000), *C*. *pseudotuberculosis* MB302 (MCNX01000000), *C*. *pseudotuberculosis* MB336 (MCNW01000000), *C*. *pseudotuberculosis* MB11 (CP013260), *C*. *pseudotuberculosis* MB14 (CP013261), *C*. *pseudotuberculosis* MB30 (CP013262), *C*. *pseudotuberculosis* MB66 (CP013263), *C*. *pseudotuberculosis* MB20 (CP016829).

All contigs smaller than 500 bp that were not aligned with their respective scaffolds were evaluated to ensure representation of genes in these genomes. The contigs were analyzed for the presence of coding DNA sequences (CDSs). The contigs with predicted CDSs were subsequently included in the files used in the comparative analysis. Although the genomes had possible gaps in their sequences, their sizes were very similar to other complete genomes of the same species [14, 16] (Table 1).

Of the seven strains described, two were responsible for severe infectious processes that culminated in the death of the animal (MB122 and MB154). The strain MB122 was isolated from the liver of an Arabian horse that was 8 years old with septicemia and had microbial growth in at least three different internal organs. The MB154 strain was isolated from an English thoroughbred that was 14 years old and also had suspected sepsis and bacterial growth in at least three internal organs. The thoroughbred did not respond to antimicrobial therapy with trimethoprim/sulfamethoxazole (TMS). Three other strains were isolated from animals with severe internal infection (MB278, MB295, and MB302). These animals recovered fully after antimicrobial treatment. In contrast, the strains MB44 and MB336 showed less severe infections without the need for antibiotic treatment (Pectoral abscesses) (Table 1).

**Fig 1:**
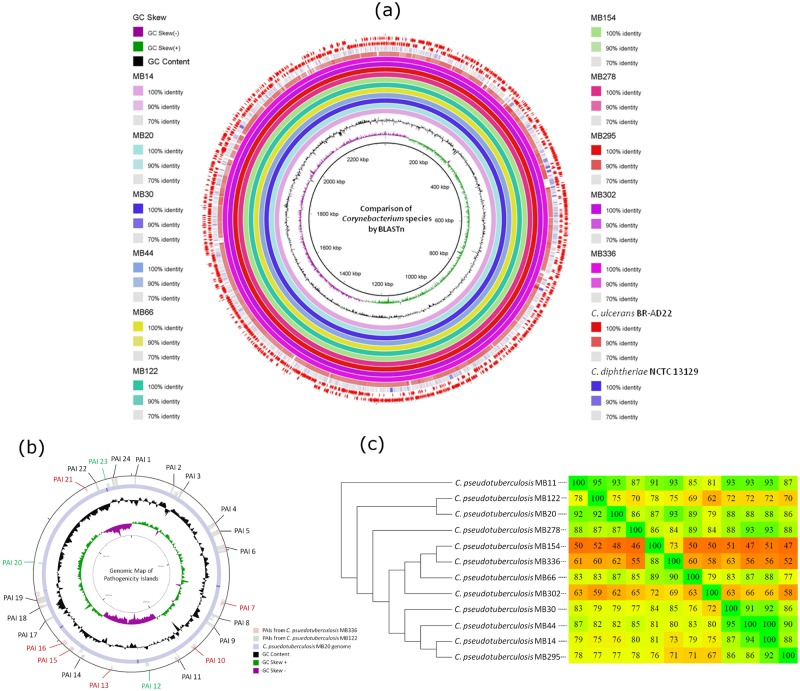
Map of the circular genomes of *C*. *pseudotuberculosis*, *C*. *ulcerans* and *C*. *diphtheriae* and the analysis of GEIs. The genomes were compared by blastn, and the percentage of identity between them was determined by the intensity of the color in the circular map. The genomes of *C*. *pseudotuberculosis* were identified only by the name of the lineage. (a) The innermost ring to the outermost is presented in this figure, as follows: the GC skew of the MB14 strain; the GC contents of the MB14 strain, the genomes of the *C*. *pseudotuberculosis* strains MB14, MB20, MB30, MB44, MB66, MB122, MB154, MB278, MB295, MB302, and MB336, and the genomes of *C*. *ulcerans* BR-AD22 and *C*. *diphtheriae* NCTC 13129. The two outermost rings comprise CDSs identified in the genome of *C*. *pseudotuberculosis* MB14. The graph displayed by GC skew is common to chromosomes that have bidirectional replication. (b) A circular genome map of the MB20 strain was constructed by comparing the position of 18 PAIs in the MB122 strain and 21 PAIs the in MB336 strain through tblastx. The PAIs shown in black were detected in both strains by GIPSy, while the PAIs in green were detected only in MB122 and the PAIs in red were detected only in MB336. A later comparison using blastn showed that the conservation of these PAIs was higher than that observed by GIPSy. (c) A dendrogram was calculated with the Neighbor-Joining model from the comparison of the nucleotide sequences of the RIs of 12 Californian isolated genomes.

Initially, a comparative ring was generated with BRIG v.0.95 software (BLAST Ring Image Generator) [25] to analyze the identity between the seven sequenced genomes using blastn. Five other genomes of the biovar *equi* from the strains isolated in California (MB20, MB11, MB14, MB30, and MB66) were also used in addition to the genomes of the phylogenetically related species *Corynebacterium ulcerans* BR-AD22 and *Corynebacterium diphtheriae* NCTC 13129. In the rings of Fig 1a, it is possible to notice that the 12 genomes of biovar *equi* showed high identities (between 90–100%) without the formation of large visible gaps. From the analysis of 16S rDNA, *C*. *ulcerans* has been described as a species of the genus *Corynebacterium* that is more closely related to *C*. *pseudotuberculosis* [26] and, consequently, showed several genomic regions of high identity with the isolates from California (Fig 1a). On the other hand, *C*. *diphtheriae* notoriously showed a lower identity level (Fig 1a).

### Insights on the genetic content of GEIs and prophages

The detection of GEIs was performed with the program GIPSy v.1.1.2 [27]. Two types of GEls were detected and analyzed in this study, Pathogenicity Islands (PAIs) and Resistance Islands (RIs). The PAIs contain a large number of virulence genes grouped into blocks that play important roles in the pathogenicity of the microorganism. In principle, eighteen PAIs were identified in six of the seven sequenced genomes (Fig 1b). Only the MB336 strain showed 21 PAIs (Fig 1b). The PAIs presenting a prediction score of "weak" were not taken into consideration. These numbers were higher than the 16 PAIs previously identified by Soares and colleagues [19]. The number of virulence factors of each sequenced genome is presented in Table 1. The *C*. *pseudotuberculosis* MB44 was the strain with a higher number of detected virulence factors.

To confirm the detection of PAIs, the genome of the strain MB122, which showed 18 PAIs, and the genome of the strain MB336, which showed 21 PAIs, were compared and analyzed by blastn with the Artemis Comparison Tool (ACT) [28]. In the graph obtained with the ACT, it is possible to observe that the genes belonging to the unique islands from MB336 were also present in the genome of the strain MB122 and other isolates from California (S1 file), which suggest that the conservation of these PAIs is higher than that observed by GIPSy. These PAIs contained important virulence factors for *C*. *pseudotuberculosis*, such as the cluster of Oligopeptide Permease (Opp) genes, which has been shown to have great importance in the pathogenicity of species, such as *Streptococcus* group A (GAS) [29] and *Bacillus thuringiensis* [30]. These findings suggest that the virulence of *equi* biovar is directly related to its high plasticity, and the genomic sequencing of new genomes may assist in determining a more robust set of virulence factors.

The number of RIs was quite variable. A total of 17 RIs was predicted for the strains MB122, MB278, and MB295, while strains MB44, MB154, MB302, and MB336 showed 16 RIs, 22 RIs, 15 RIs and 19 RIs, respectively. The numbers of genes encoding proteins involved in antibiotic resistance are described in Table 1. Interestingly, the strains with the highest number of RIs had a lower number of detected resistance genes. Among them is the MB154 strain, which causes a severe infection with septicemia. Despite these genotypic differences described above, Rhodes and colleagues [31] found no significant phenotypic differences in the antimicrobial resistance profiles of strains of *C*. *pseudotuberculosis* isolated over 16 years (1996–2012).

The nucleotide sequences of RIs for the seven sequenced genomes and other five genomes of bacteria isolated in California were recovered with Artemis software and analyzed using fragmented all-against-all comparison with the Gegenees software v. 2.2.1 [32]. The comparative results of Gegenees are presented as a heatmap containing the percentage of identity between the samples. This heatmap was analyzed with SplitsTree4 v.4.14.2 [33] for the calculation of a Neighbor-Joining dendrogram (Fig 1c). The strains with the largest number of islands (MB154 and MB336) were more closely related and grouped in a distinct clade (Fig 1c). These results indicated, despite the lower number of resistance genes for these two strains, the genes were clustered into a greater number of islands and, therefore, were acquired from a larger number of horizontal gene transfer events when compared to the other strains, generating greater variability. However, this gained genetic content was not enough to influence a change in the antimicrobial resistance profile [31]. A high number of genes related to resistance against antibiotics in *C*. *pseudotuberculosis* biovar *equi* were detected including beta-lactamase enzymes, membrane permeases, efflux pumps and others (S1 Table).

**Fig 2:**
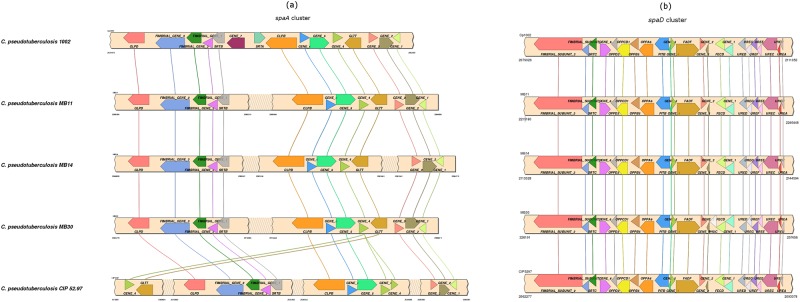
Synteny analysis of *C*. *pseudotuberculosis* pili gene clusters. From top to the bottom of the figure the genome of strains 1002, MB11, MB14, MB30 and CIP 52.97 are shown. Genes are represented by arrows of different color. Genes without standardized names are identified as gene_1, gene_2, and so on. Conserved genes are connected by lines. (a) *spaA* cluster. (b) *spaD* cluster.

Two predicted PAIs carry the gene clusters *spaA* and *spaD* responsible for the expression of pili proteins (Fig 2). A Pilus is a filamentary cell surface structure that provides adhesive and invasive functions to the cell of the pathogen [34]. Genes of both clusters were obtained from the genome of *C*. *pseudotuberculosis* 1002 biovar *ovis*. Genes were compared by blastn with the nucleotide sequence of the complete genomes of strains 1002 (biovar *ovis*), MB11 (biovar *equi*, caused ulcerative lymphangitis), MB14 (biovar *equi*, infection with internal abscess formation), MB30 (biovar *equi*, infection with external abscess formation), and CIP 52.97 (the outmost taxon of biovar equi). The comparison was performed using the web tool Simple Synteny [35]. Those genes with a minimum coverage cutoff of 50% were identified and were represented in Fig 2. The *spaD* cluster showed high conservation in all strains analyzed (Fig 2), while *spaA* cluster presented some genomic rearrangements. Strains of the biovar equi showed a deletion in the region that should contain the sortase gene *srtA* (Fig 2). A gene prediction followed by manual curation identified *srtA* as a pseudogene in all strains of biovar equi. Sortase enzymes are essential for the assembly and anchoring of pilus structure in the bacterial cell wall [36], and this type of arrangement has been demonstrated by Soares and colleagues for 16 genomes of *C*. *pseudotuberculosis* [19]. All the three strains of biovar equi analyzed have the same arrangement of pili genes (Fig 2) which potentially rejects the possibility that these genes may have a major influence on the type of infection caused by the pathogen.

**Fig 3:**
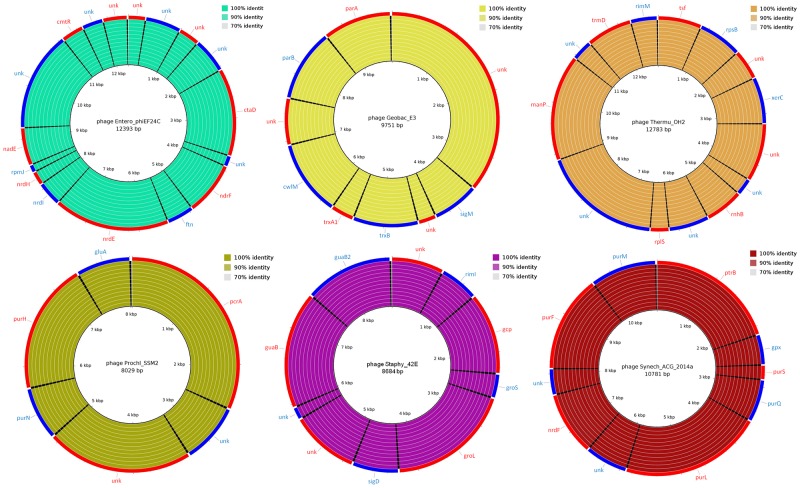
Circular map of *C*. *pseudotuberculosis* prophages identified with the online tool PHAST. Each circular map represents an identified prophage. The outermost ring describes the genes of each prophage that were interspersed **(**colored in blue and red). The acronym "unk" identifies uncharacterized proteins. The innermost ring to the outermost represents the results of blastn for the genomes of *C*. *pseudotuberculosis* MB11, MB14, MB20, MB30, MB44, MB66, MB122, MB154, MB278, MB295, MB302, and MB336. The level of identity, nearly 100% for all genes, indicated high conservation of these prophages in the 12 analyzed genomes.

In addition to the detection of PAIs and RIs, the prophages were identified with the online tool PHAST [37]. Six incomplete prophages were detected containing both bacterial and phage genes that were interleaved (Fig 3). These regions showed high conservation both structurally (position of genes) and functionally (high identity) in the twelve genomes of Californian isolates (Fig 3). Of the 66 genes detected in prophages, 24 were uncharacterized proteins (approximately 36% of the total genes).

One of the conserved prophages was related to phage E3 of the *Geobacillus* sp. (Fig 3), an extremophile genus commonly isolated from soil samples [38], in the main environmental habitat of *C*. *pseudotuberculosis*. This prophage contains the sequence of the alternative sigma factor M (*sigM*), which is involved in bacterial adaptation to changes in the extracellular environment, such as oxidative stress. Interestingly, downstream of *sigM* in prophage E3 we found the *trxB* and *trxA1* genes, which encode thioredoxin enzymes that have an antioxidant function and are regulated by *sigM*. Combating oxidative stress is necessary for the persistence of *C*. *pseudotuberculosis* in the host organism because the bacteria behaves as an intracellular parasite. After phagocytosis, they are exposed to the harsh environment of the phagosome, which contains a high concentration of harmful oxidizing agents to the cell of the invading pathogen.

In a second incomplete prophage, OH2, the *xerC* gene coding for a phage integrase containing the tyrosine recombinase domain was detected (Fig 3). The recombinases are involved in the process of excision/integration of mobile genetic elements in bacterial genomes and thus are responsible for genome rearrangements and acquisition and/or gene silencing. Nevertheless, the *xerC* gene is in a genomic region highly conserved without visible genomic rearrangements (S1 file).

Downstream *xerC* gene is a non-characterized protein containing a domain of the M23 family of metallopeptidases, a group of endopeptidases that catalyze the hydrolysis of the bacterial peptidoglycan, leading to cell lysis [39]. These proteolytic enzymes are important virulence factors of the pathogen because they must overcome other bacterial cells of the host microflora to succeed in the invasion process during infection. The presence of a metallopeptidase, besides a recombinase gene, is conserved even in temporally and spatially distant genomes such as *C*. *pseudotuberculosis* CIP 52.97, which indicates an ancient and widespread event of horizontal gene acquisition within the genus *Corynebacterium*.

### Phylogenomic inference and SNP-based clustering

**Fig 4:**
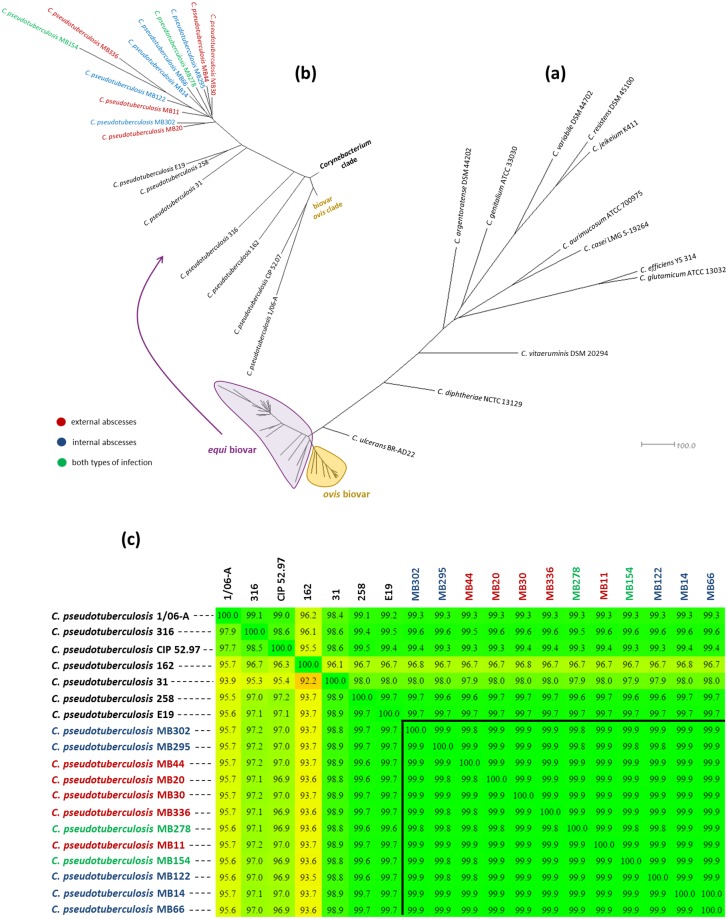
Phylogenomic analysis of the *Corynebacterium* genus. (a) The entire Neighbor-Joining tree containing the *Corynebacterium* RefSeq genomes, demonstrating the closeness between the species *C*. *diphtheriae*, *C*. *ulcerans* and *C*. *pseudotuberculosis*. This latter species comprises two distinct clades composed of strains from biovar *ovis* (marked in orange) and biovar equi (marked in purple). (b) An expanded view of the biovar *equi* tree. Strains isolated in California (in color) are closely related to each other. The strains CIP 52.97 and 1/06-A formed the outermost clade of biovar *equi*. The colors of the names from strains isolated in California distinguish the types of infection caused by the pathogen. (c) Dendrogram calculated with the Neighbor-Joining model from heatmap values generated in the Gegenees program. The values shown in the heatmap indicate the percentage of similarity between the analyzed genomes. The name of the species isolated in California is colored according to the type of infection caused by the pathogen.

Two approaches were performed to evaluate the genomic and evolutionary similarity of the species *C*. *pseudotuberculosis*. The first used PGAP V.1.11 [40] software, which took into account the core genome to calculate dendrograms based on the Neighbor-Joining model (Fig 4a and 4b). The unrooted tree of Fig 4a indicates a high distinction between the species *C*. *pseudotuberculosis*, and the rest of the environmental and pathogenic species of the genus *Corynebacterium*. *C*. *vitaeruminis*, *C*. *diphtheriae*, and *C*. *ulcerans* converged, respectively, to form an evolutionary path to the species *C*. *pseudotuberculosis*. *C*. *ulcerans*, therefore, is the species of the genus most closely related to the species *C*. *pseudotuberculosis*, corroborating the phylogenetic analysis of 16S rDNA [26]. The nucleotide identity between these two species can be observed in the rings of Fig 1.

No phenetic distinction between species that cause internal and external abscesses could be observed within the clade of *C*. *pseudotuberculosis* biovar *equi*. However, it is evident that there was greater proximity between the strains isolated in California in comparison with the other species from biovar *equi* (Fig 4a and 4b). It is worth noting that the strains CIP 52.97 and 1/06-A are more closely related to each other and form an outer clade, distinct from other strains of the biovar (Fig 4b). The CIP 52.97 strain is the one with greater temporal distance from the other analyzed strains being isolated in 1952 and kept in the Pasteur Institute Collection [41].

In the second approach, only the strains belonging to the biovar *equi* were compared using Gegenees v.2.2.1 software. In this analysis, the genomes were fragmented in blocks of 200 bp, and these blocks were compared all-against-all with blastn, generating a heatmap based on standardized blast values (Fig 4c). From these values, a Neighbor-Joining dendrogram was calculated with the SplitsTree4 software, as described above for the RI analyses. As shown in Fig 4b, the analysis with Gegenees indicated CIP 52.97 and 1/06-A as the outer strains of biovar *equi*. It was also observed that the strains MB154 and MB122, which caused more severe infectious conditions and challenging treatment (Table 1), were closely related in both phylogenomic analyses. However, no genome region of these two strains significantly differed from the genomes of other strains that caused less severe infections with external abscess formation (S1 Fig).

As expected, the topography of the dendrogram calculated only for the RIs was quite different from that observed in the dendrogram calculated for the entire genomic sequence. While the dendrogram of RIs grouped species with similar clinical history, the second dendrogram distinguished the species isolated in California from the rest of the strains of biovar *equi*, analogous to that observed in the tree shown in Fig 4a.

A clustering analysis based on the SNPs detected in the core genome of genus *Corynebacterium* was performed. Maximum likelihood method was used to calculate the tree. As the gene density in prokaryotes is very high, SNPs can drastically change protein structures and consequently modify the bacterial phenotype. However, the topography of the SNP-based tree was similar to that constructed using a Neighbor-joining method based on the gene content of the core genome (S2 Fig). These results emphasize the phenetic and cladistic distance between the isolates of California and the other strains of biovar *equi*.

### Pangenome evaluation of biovar *equi*

**Fig 5:**
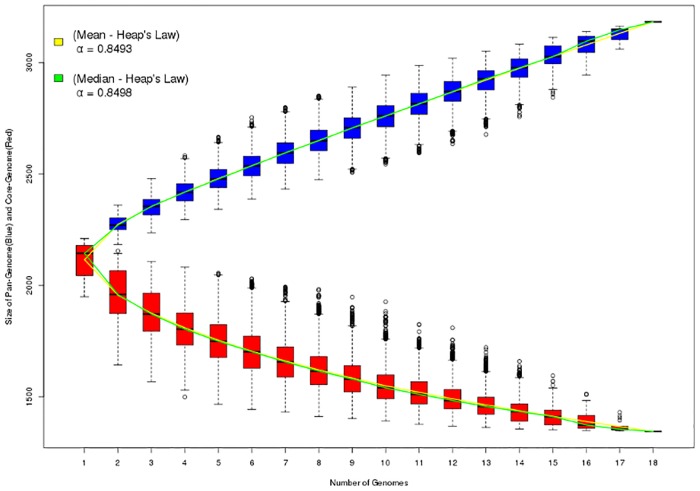
Prediction of the core genome and pangenome of eighteen genomes of *C*. *pseudotuberculosis* biovar *equi*. The graphic bars represent the number of core genes (red bars) and pangenome (blue bars) for *n* combinations of the analyzed genomes. The dotted lines indicate the standard deviation of these combinations and white circles outside the dotted lines indicate discordant values of the analysis. The calculation of the pangenome and core genome size showed very similar values using either the mean (yellow line) or median (green line) of the possible combinations.

The α value of Heap's Law was calculated based on the results of the pangenome obtained in PGAP V.1.11 to characterize the genomic plasticity level of biovar *equi*. Eighteen genomes available in the NCBI database (seven of them sequenced in this study) were used. Previously, Soares and colleagues [19] demonstrated that *C*. *pseudotuberculosis* had an open pangenome (α value = 0.89) and the *equi* biovar had higher plasticity compared to biovar *ovis*. The analysis of Soares and colleagues [19] was performed with the EDGAR software [42]. The pangenome graph shown in Fig 5 indicates that, even after a significant increase in the number of sequenced genomes from biovar *equi*, the value of α = 0.849 indicated that this pangenome was still open. This means that the sequencing of new genomes will possibly add new genes not described in this pangenome.

**Fig 6:**
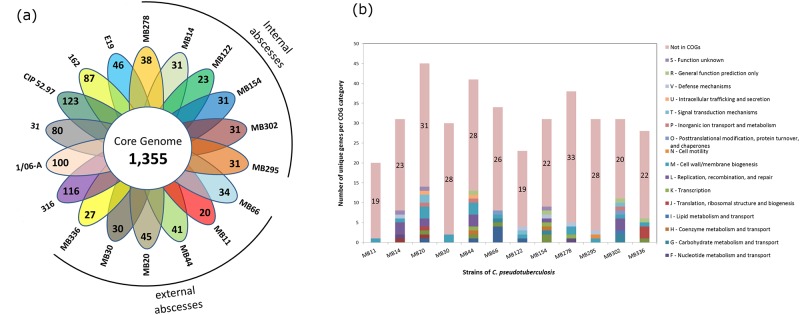
Graphical representations of the pangenome characteristics of biovar equi. (a) Flower graph representing the number of core and accessory genes for each strain of biovar *equi*. The strains are listed by their names along with each set of singletons. Those strains causing internal or external abscesses are indicated by black lines. (b) Bar graph of singletons classified in COG categories. The numbers in the bars indicate the amount of genes not classified in COGs.

The pangenome of biovar *equi* consists of 3,183 genes, with a core genome of 1,355 genes, which was less than half of the genes described in the pangenome (approximately 42.5%) and is a result that enhances the high genetic variability of this biovar (Fig 6a). This percentage of the core genome is one of the lowest reported in the literature compared with the pangenomes of other bacterial species, such as *Escherichia coli* that has 44% of core genome, *Pseudomonas syringae* with 64%, *Streptococcus pneumoniae* with 74%, and *Listeria monocytogenes* with 80%, among others [43–46]. It is possible to note in the flower graph of Fig 6a that the number of singletons was highest in strains CIP 52.97, 1/06-A and 316. However, the number of singleton genes is quite low in the remaining genomes (Fig 6a); e.g. singleton genes of MB44 correspond to only 2% of the total predicted CDSs. Thus, doubletons (genes shared by two strains) and other accessory genes contribute more significantly than singletons to the high variability of the biovar. The singleton genes were classified into categories of the Clusters of Orthologous Groups (COGs) using the Batch Web CD-Search Tool (https://www.ncbi.nlm.nih.gov/Structure/bwrpsb/bwrpsb.cgi). Most genes were not classified into COGs (Fig 6b) and therefore their biological functions are unknown. The most represented COG categories were M—Cell wall/membrane biogenesis and L—replication, recombination and repair (Fig 6b).

Some substantial differences were observed between the genomes of strains isolated in California and the genomes of strains CIP 52.97 and 1/06-A, which were phylogenetically more distant within biovar *equi*. Therefore, the strains with complete genomes, MB14, CIP 52.97 and *C*. *diphtheriae* NCTC 13129, were compared with blastn, and the results were visualized with ACT (S1 file). Five insertion-regions were detected in the genomes of isolates from California. It is also possible that these regions were deleted from the genome of CIP 52.97. One of these regions was found upstream of the gene *mas* (S3 Fig). This gene is a virulence factor encoding the enzyme mycocerosic acid synthase, which is responsible for the production of phenolic glycolipids. These substances are the main components of the waxy material of the cell wall of mycobacteria [47] and are largely responsible for its pathogenicity. The insertion/deletion observed contained six genes that encode uncharacterized proteins and a seventh gene coding for a protein with a resolvase domain, which is very common in endonucleases involved in DNA recombination. Interestingly, *C*. *diphtheriae* has a prophage sequence of approximately 15 Kbp inserted in the same genome site. This sequence showed no similarity to the sequence found at the same location for the isolates from California. Therefore, this is probably a common recombination site of *Corynebacterium* genus, where the events of horizontal gene transfer that occasionally happened in *C*. *pseudotuberculosis* and *C*. *diphtheriae* were evolutionarily independent.

Five genes of uncharacterized proteins were analyzed with the online tool InterProScan [48] to detect conserved protein domains. Two CDSs had no predicted domains. The other three had a catalytic domain of phage integrase (IPR013762), a P-loop containing nucleoside triphosphate hydrolase (IPR027417) and a HNH nuclease domain (IPR003615). The latter is commonly found in bacteriocins, which are proteins secreted by microorganisms that have antimicrobial activity against closely related bacterial strains [49]. Thus, it is probably an important factor that contributes to the long persistence of *C*. *pseudotuberculosis* in environments outside the host, such as soil [50].

The identification of an uncharacterized protein containing a phage integrase domain indicates that *C*. *pseudotuberculosis* genomes probably have a larger number of incomplete prophages than just the six identified by PHAST (Fig 3). Moreover, these results emphasize the need to analyze the uncharacterized proteins of *C*. *pseudotuberculosis*, as these may play important roles in the pathogenesis and persistence of the bacteria in the environment or the host.

In addition to the features described above, a peptidase S8A classified in the family of subtilisin-like serine protease (SRSP) was found upstream of gene *mas*, which has been previously described as an important virulence factor in *C*. *pseudotuberculosis* FRC41 [21], one of the few strains isolated from human hosts. In the strain CIP 52.97, this virulence factor is a pseudogene due to the presence of a deletion in the genome and, hence, is probably non-functional, while in the MB14 strain this CDS is complete, without the presence of frameshifts (S3 Fig). Five other serine proteases were found in the genomes of isolates from California, which is a number larger than that described for the human strain FRC41. These findings demonstrated the vast repertoire of virulence factors present in the genomes of the Californian isolates.

## Conclusions

In this study, seven new genomes of the re-emerging pathogen *C*. *pseudotuberculosis* biovar *equi* were sequenced and assembled in high-quality scaffolds with a size of 2.3 Mbp. These genomes showed molecular characteristics that were very similar to the complete genomes of other strains from biovar *equi*.

Comparative genomics results demonstrated that *C*. *pseudotuberculosis* carries an extensive repertoire of virulence factors and resistance genes in its genome, e.g., peptidase enzymes, phage integrases, recombination endonucleases, micoside synthesis enzymes, bacteriocins with antimicrobial activity, beta-lactamases, antibiotic efflux pumps and other molecular factors that shape the host-pathogen interaction process. The pangenome demonstrated that the genetic variability of the biovar was greater than previously thought. No genotypic characteristic that distinguished the strains according to the type of infection has been identified. In fact, regardless of the type of infection, isolates of California showed greater genomic similarity and phylogenetic proximity with each other than with the rest of the strains of biovar *equi*. The CIP 52.97 and 1/06-A strains were related less to the remaining strains of the biovar.

The vast majority of singletons detected in the pangenome of *C*. *pseudotuberculosis* biovar *equi* were uncharacterized proteins and more detailed analysis of at least three of these proteins into the genome of strain MB14 identified probable new factors that contribute to the virulence of the species. This emphasizes the importance of characterizing proteins with unknown function in bacterial pathogens.

## Methods

### Human or animal subjects and/or tissue or field sample

The study was not submitted to any committee. The experiments do not require the approval of committees, as they involve the genome sequencing of previously isolated bacterial strains.

### Data collection, isolation, and cultivation

The *C*. *pseudotuberculosis* strains described in this study were previously isolated from horses in the state of California, USA. Information about the animal host, date and site of isolation of the microorganism, and the clinical condition of the horses can be observed in S1 Table. Table 1 presents this information in summary form.

Abscess samples were obtained and used for the cultivation of *C*. *pseudotuberculosis* in Brain-Heart Infusion (BHI). After isolation, the bacteria were maintained in 25% glycerol at -80°C. For extraction of the genomic DNA, the strains were also grown in BHI medium at 37°C with shaking. The extraction occurred when the optical density of the culture reached the mid-log phase of bacterial growth according to the procedures described by Pacheco and colleagues [51] for clinical isolates of *C*. *pseudotuberculosis*. To evaluate the DNA extraction protocol, an aliquot of DNA was migrated in a 1% agarose gel.

### Sequencing, assembly and annotation of genomes

The seven genomes in this study were sequenced with the Ion Torrent PGM platform (Thermo Fisher Scientific) using the chip 318 according to the manufacturer's protocol. The quality of sequenced reads was visualized with FastQC software (http://www.bioinformatics.babraham.ac.uk/projects/fastqc/) and, when necessary, was trimmed and filtered using a Phred score of 20. The assembly was performed with MIRA 4 [52] software and the redundancies of the contigs were removed with the SeqMan Pro tool of the Lasergene software (DNASTAR). Most of the remaining gaps were manually closed using local blastn or using GapBlaster software, which uses a reference genome to map sequenced reads to generate sequences that closed the gap. The complete genome of *C*. *pseudotuberculosis* 316 was used as a reference.

The final few contigs were ordered in software MAUVE using *C*. *pseudotuberculosis* MB11 as the reference genome because its assembly was confirmed by the *in vitro* optical map. The high-quality scaffolds were submitted to the web software Pannotator [53] for automatic annotation using the *C*. *pseudotuberculosis* 316 as the reference genome. Subsequently, the predicted CDSs were manually cured with Artemis v.14.0.0 software [24] to meet the gene annotation criteria of UniProt (http://www.uniprot.org) and to remove pseudogenes formed due to errors of the sequencing platform. The level of genomic similarity between the sequenced isolates was assessed by blastn and visualized as a circular map created with the program BRIG v.0.95 [25].

The Whole Genome Shotgun project of each genome has been deposited at GenBank under the accession numbers MCOC00000000, MCOB00000000, MCOA00000000, MCNZ00000000, MCNY00000000, MCNX00000000, and MCNW00000000. The versions described in this paper are versions MCOC01000000, MCOB01000000, MCOA01000000, MCNZ01000000, MCNY01000000, MCNX01000000, and MCNW01000000.

### Identification and analysis of GEIs and prophages

The PAIs and RIs were identified with GIPSy v1.1.2 software [27]. *C*. *glutamicum* ATCC 13032 was used as a non-pathogenic strain of reference. The complete lists of virulence factors, resistance genes, locations and composition of PAIs and RIs can be viewed in the S1 table. The nucleotide sequence of each RI was recovered with Artemis v.14.0.0 software. The RIs of each strain were grouped into a multi-fasta and compared all-against-all using Gegenees v.2.2.1 software [32]. As a result of the comparison, a heatmap containing the percentage similarity of these islands was generated, which was subsequently used for calculating a Neighbor-Joining dendrogram with SplitsTree4 v.4.14.2 software [33].

The sequence of the genes from each PAI was also recovered using Artemis. These sequences were compared with the genome of the strain MB20 using tblastx in CGView Server [54] to create a circular map of the location of these PAIs. The identification of these PAIs was subsequently confirmed by blastn between the genomes of MB122 and MB336, which were observed in the ACT software [28]. A database of pili genes was created from the genome of *C*. *pseudotuberculosis* 1002. Those genes were compared to the nucleotide sequences of strains 1002, MB11, MB14, MB30, and CIP 52.97 using blastn with a minimum coverage cutoff of 50%. Analysis was performed in the web tool Simple Synteny [35].

The identification of prophages in the genomes was performed with the web tool PHAST (Phage Search Tool) [37]. A database containing all the genes identified in these prophages was generated to evaluate their conservation in the analyzed genomes. These genes in.fasta format were compared with the nucleotide sequences of the twelve genomes of Californian isolates generating a circular map in BRIG software.

### Phylogenomic analysis and SNP-based clustering of *Corynebacterium*

The genomes of twelve reference species (RefSeq) from the *Corynebacterium* genus were recovered from the National Center for Biotechnology Information (NCBI) database in the GenBank file format. These files were used to determine the phylogenetic relationship among the isolates from California with the rest of the species from the *Corynebacterium* genus. All available genomes of biovar *ovis* of *C*. *pseudotuberculosis* were also used in the analysis. The construction of the phylogenomic tree was performed using two approaches.

At first, the construction of a phylogenetic tree in the Neighbor-Joining model was performed using the core genome of the genus calculated in PGAP v.1.11 [40]. The tree was displayed and edited in the SplitsTree4 program. In the second approach, only the 19 genomes of the biovar *equi* were used. Therefore, the nucleotide sequences of these 19 genomes were compared all-against-all using blastn in the Gegenees program. For comparison, we used the protocol of high accuracy with the fragmentation of genomes in 200 bp blocks with a step size of 100 bp. The similarity between the genomes was plotted as a heatmap containing percentage identity values. These values were used to construct a Neighbor-Joining dendrogram in the SplitsTree4 program.

A SNP-based genotyping analysis was performed using PGAP software. SNPs of the core genome were used to build a Maximum Likelihood tree that was subsequently analyzed in the SplitsTree software.

### Pangenome analysis of biovar *equi*

Initially, the pangenome of the biovar was calculated with the PGAP V.1.11 program using the GF method with the program’s default parameters. The data from 1.Orthologs_cluster.txt file were used for the construction of a flower graph containing information about the core and singletongenes. Subsequently, the 2.CDS.variation.txt file was used for the building of the Heap's Law graph using a script in R. The comparison of virulence factors and resistance genes contained in the genomes was performed using the ACT program. A sequence database of singleton genes from strains of *C*. *pseudotuberculosis* biovar *equi* was obtained using a script in perl developed by our laboratory named getFastaFromOrthologs.pl. The script uses the 1.Orthologs_cluster.txt file to identify singletons and extract their amino acid sequences from the.pep files. These genes were classified in Cluster of Orthologous Groups (COG) using the Batch Web CD-Search Tool (https://www.ncbi.nlm.nih.gov/Structure/bwrpsb/bwrpsb.cgi).

## Supporting Information

S1 File Archives of *C*. *pseudotuberculosis* MB14, *C*. *pseudotuberculosis* CIP 52.97, and *C*. *diphtheria* NCTC 13129 for analysis in the ACT program. The blastn comparison files are named as strain_vs_strain. For best viewing, the GenBank files are provided, containing all predicted CDSs in the genome.
(ZIP)

S1 Table Tables containing the results for detection of RIs and PAIs using the Gipsy program. All tables are grouped into a single file. The first two contain information for each identified island, while the remaining tables contain a description of the virulence genes (virulenceDB) or antibiotic resistance genes (resistanceDB). The genes not identified as virulence or resistance factors were identified with No Hits Found.
(XLSX)

S1 Fig Graphical representation of the similarity between the genomes of strains MB154 and MB122 compared with the genomes of strains that caused external abscesses (MB44, MB20, MB30 and MB336). Horizontal black bars indicate the size of the MB154 genome. The three vertical black bars indicate regions of gap. The vertical red bars determine the level of divergence between the MB154 genome and the other genomes of Californian isolates, according to the color displayed in the lower right corner. It was not possible to identify unique regions in the genome of the most virulent strains MB154 and MB122. The graph was obtained with the Gegenees program and was calculated from the all-against-all comparison analysis.
(TIF)

S2 Fig SNP-based clustering of *Corynebacterium* genus calculated with PGAP. The tree was calculated using Maximum Likelihood method. Tree was analyzed in SplitsTree software. To better visualize the topography of the tree, bacterial names were abbreviated as follows: *C*. *pseudotuberculosis* (Cp); *C*. *ulcerans* (Cu); *C*. *diphtheriae* (Cd); *C*. *vitaeruminis* (Cv); *C*. *aurimucosum* (Cauri); *C*. *casei* (Cc); *C*. *genitalium* (Cgen); *C*. *argentoratense* (Carg); *C*. *glutamicum* (Cg); *C*. *efficiens* (Ce); *C*. *variabile* (Cvari); *C*. *resistens* (Cr); *C*. *jeikeium* (Cj). Abbreviations are followed by the names of the strains. The *C*. *pseudotuberculosis* cluster is highlighted in orange. Strains isolated at California were marked with a dotted line.
(TIF)

S3 Fig Description of an insertion region in the genome of strain MB14. Horizontal gray bars represent the genomes of *C*. *pseudotuberculosis* MB14, *C*. *pseudotuberculosis* CIP 52.97, and *C*. *diphtheria* NCTC 13129. A discussion about the observed genetic content was guided by the position of gene *mas*.
(TIF)
