# Supplementary figures and images for: Assessing the Genotypic Differences between Strains of Corynebacterium pseudotuberculosis biovar equi through Comparative Genomics
Source: PLoS One. 2017 Jan 26;12(1):e0170676. doi: 10.1371/journal.pone.0170676 (PMC5268413; doi:10.1371/journal.pone.0170676)

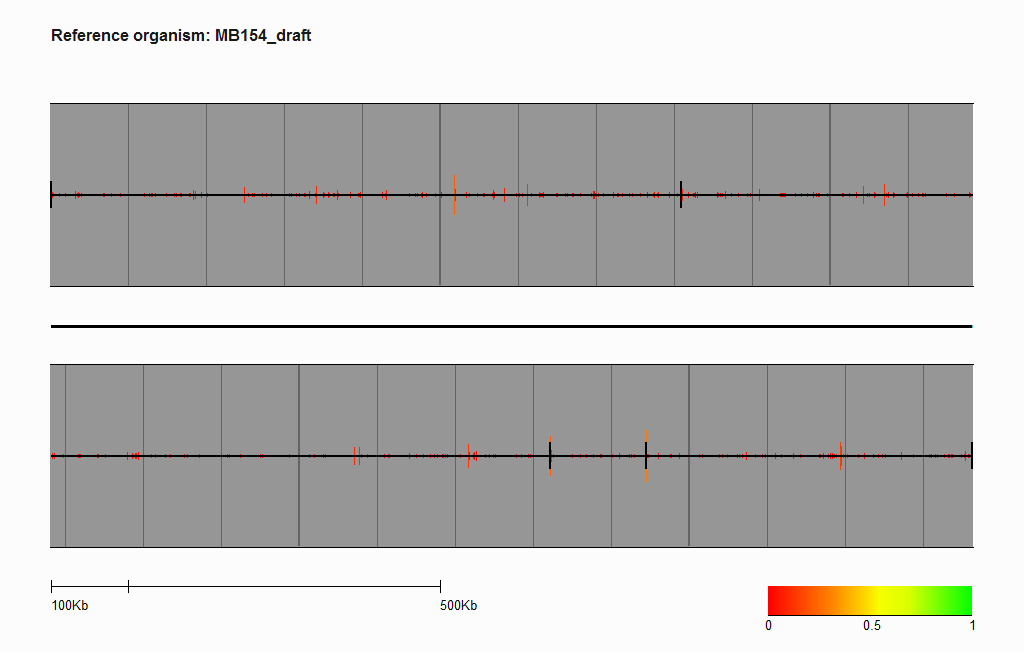

Supplement: S1 Fig — Horizontal black bars indicate the size of the MB154 genome. The three vertical black bars indicate regions of gap. The vertical red bars determine the level of divergence between the MB154 genome and the other genomes of Californian isolates, according to the color displayed in the lower right corner. It was not possible to identify unique regions in the genome of the most virulent strains MB154 and MB122. The graph was obtained with the Gegenees program and was calculated from the all-against-all comparison analysis. (TIF) [file pone.0170676.s003.tif]

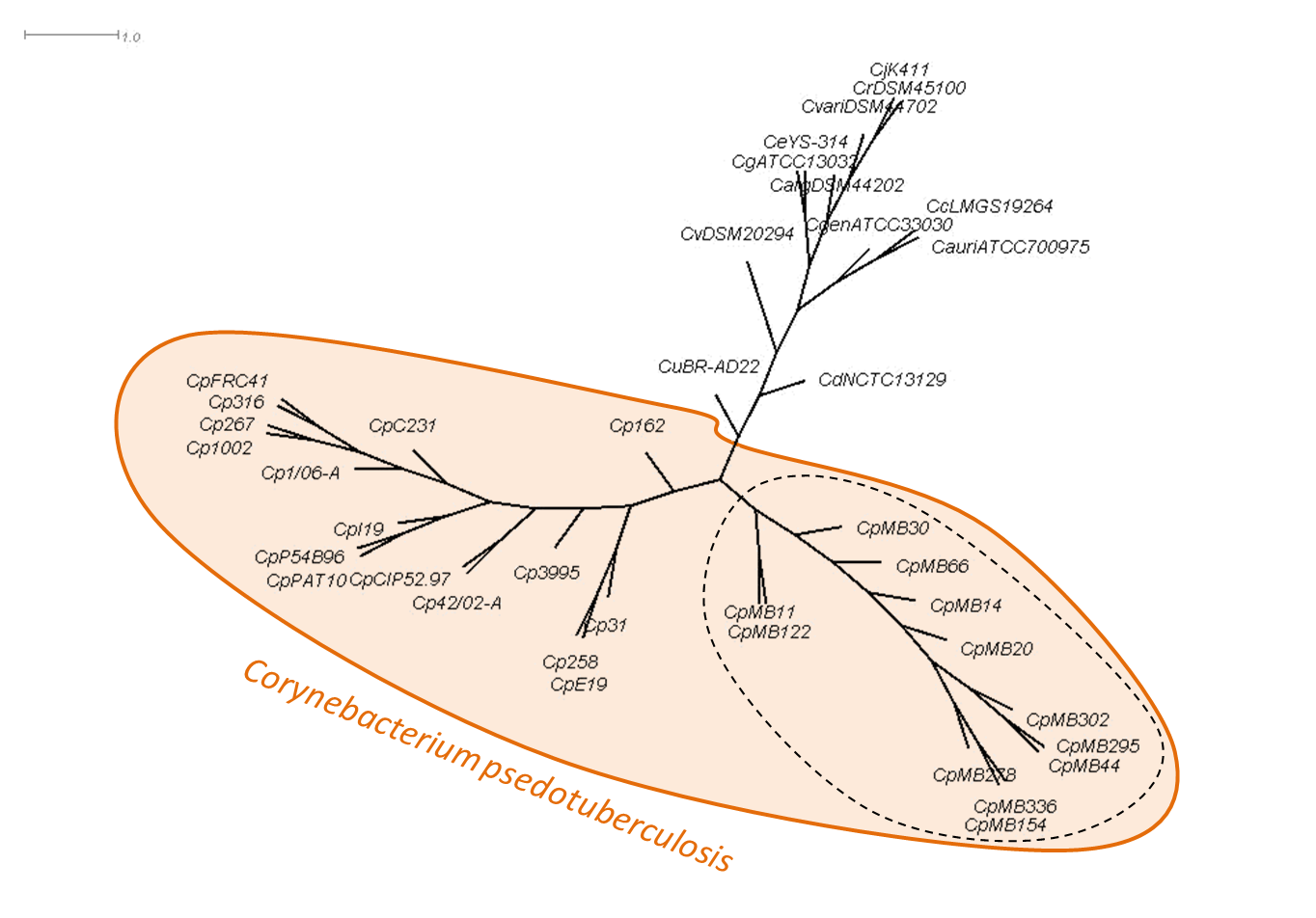

Supplement: S2 Fig — The tree was calculated using Maximum Likelihood method. Tree was analyzed in SplitsTree software. To better visualize the topography of the tree, bacterial names were abbreviated as follows: C. pseudotuberculosis (Cp); C. ulcerans (Cu); C. diphtheriae (Cd); C. vitaeruminis (Cv); C. aurimucosum (Cauri); C. casei (Cc); C. genitalium (Cgen); C. argentoratense (Carg); C. glutamicum (Cg); C. efficiens (Ce); C. variabile (Cvari); C. resistens (Cr); C. jeikeium (Cj). Abbreviations are followed by the names of the strains. The C. pseudotuberculosis cluster is highlighted in orange. Strains isolated at California were marked with a dotted line. (TIF) [file pone.0170676.s004.tif]

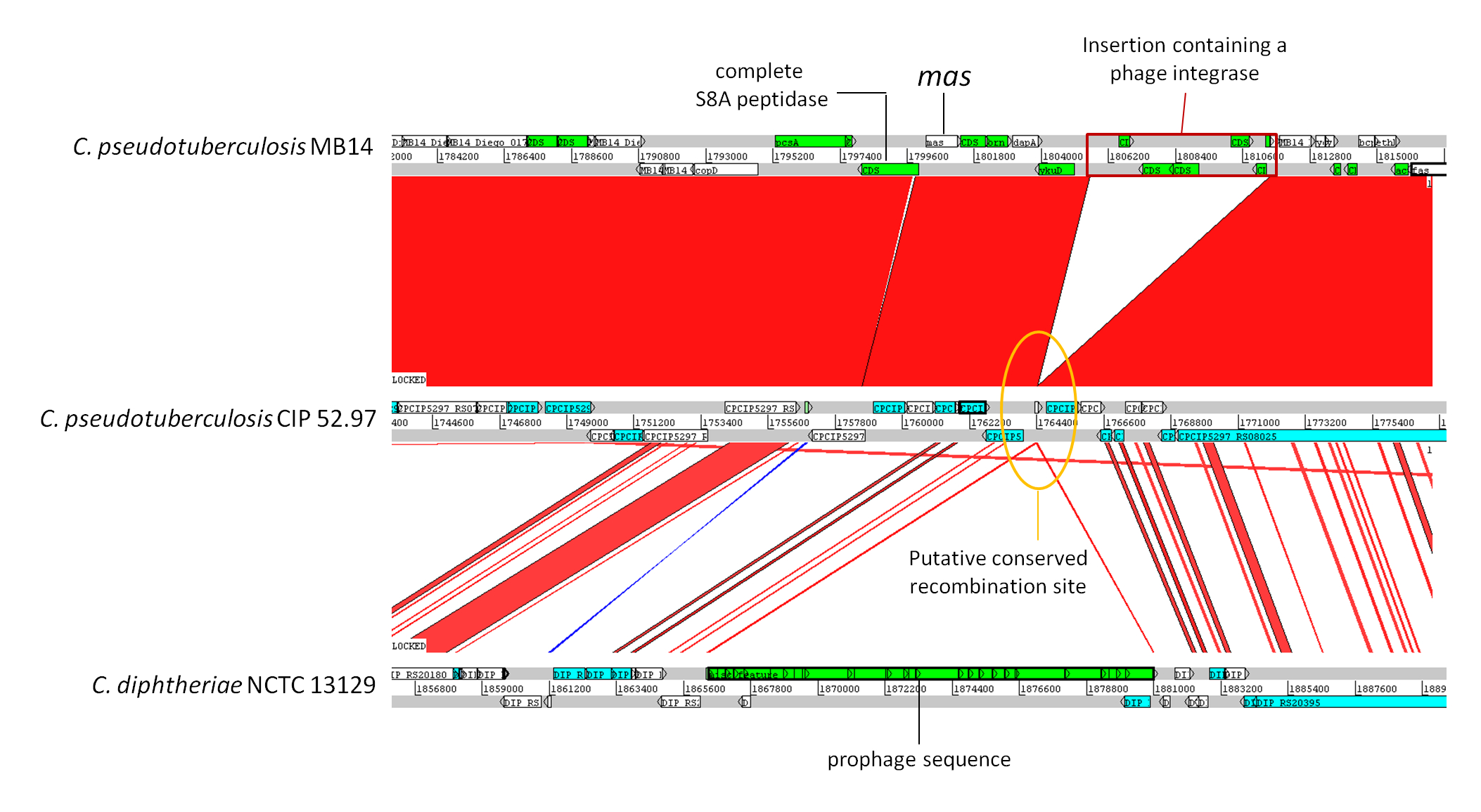

Supplement: S3 Fig — Horizontal gray bars represent the genomes of C. pseudotuberculosis MB14, C. pseudotuberculosis CIP 52.97, and C. diphtheria NCTC 13129. A discussion about the observed genetic content was guided by the position of gene mas. (TIF) [file pone.0170676.s005.tif]
